# L-Leucine Improves Metabolic Disorders in Mice With *in-utero* Cigarette Smoke Exposure

**DOI:** 10.3389/fphys.2021.700246

**Published:** 2021-07-01

**Authors:** Yunxin Zeng, Taida Huang, Nan Wang, Yi Xu, Chunhui Sun, Min Huang, Chun Chen, Brian G. Oliver, Chenju Yi, Hui Chen

**Affiliations:** ^1^Research Center, The Seventh Affiliated Hospital of Sun Yat-sen University, Shenzhen, China; ^2^Faculty of Science, School of Life Sciences, University of Technology Sydney, NSW, Australia; ^3^Respiratory Cellular and Molecular Biology, Woolcock Institute of Medical Research, Sydney, NSW, Australia

**Keywords:** maternal smoking, glucose intolerance, insulin, fat mass, leucine

## Abstract

**Objectives:** Maternal cigarette smoke exposure (SE) causes intrauterine undernutrition, resulting in increased risk for metabolic disorders and type 2 diabetes in the offspring without sex differences. L-leucine supplementation has been shown to reduce body weight and improve glucose metabolism in both obese animals and humans. In this study, we aimed to determine whether postnatal L-leucine supplementation in female offspring can ameliorate the detrimental impact of maternal SE.

**Methods:** Female Balb/c mice (6-week) were exposed to cigarette smoke (SE, 2 cigarettes/day) prior to mating for 5 weeks until the pups weaned. Sham dams were exposed to air during the same period. Half of the female offspring from the SE and SHAM dams were supplied with L-leucine via drinking water (1.5% w/w) after weaning (21-day) for 10 weeks and sacrificed at 13 weeks (adulthood).

**Results:** Maternal SE during pregnancy resulted in smaller body weight and glucose intolerance in the offspring. L-leucine supplement in Sham offspring reduced body weight, fat mass, and fasting blood glucose levels compared with their untreated littermates; however somatic growth was not changed. L-leucine supplement in SE offspring improved glucose tolerance and reduced fat mass compared with untreated littermates.

**Conclusions:** Postnatal L-leucine supplement could reduce fat accumulation and ameliorate glucose metabolic disorder caused by maternal SE. The application of leucine may provide a potential strategy for reducing metabolic disorders in offspring from mothers who continued to smoke during pregnancy.

## Introduction

Tobacco smoking is a global public health risk. According to the WHO, in 2016, the smoking rate in females over 15 years old was 20.7% in Europe, 12.4% in America, and 1.9% in China, while the second-hand smoking rate in females was as high as 46.9% in China (Ding et al., 2016; World Health Organization, 2019). Maternal cigarette smoke exposure (SE), including direct and second-hand smoking during pregnancy, is a major cause of intrauterine undernutrition resulting in several adverse health outcomes, including preterm birth, low birth weight, and catch-up growth in childhood (Collaco et al., 2017; Wagijo et al., 2017). In addition to these short-term adverse effects, offspring from mothers exposed to cigarette smoke during pregnancy are also more likely to develop metabolic disorders in adulthood, such as glucose intolerance, type 2 diabetes, fatty liver changes, dyslipidemia, and cardiovascular disease (Mendez et al., 2008; Gorog et al., 2011; La Merrill et al., 2015).

Around 463 million people suffering from diabetes, which is one of the greatest public health challenges in both developed and developing countries (Saeedi et al., 2019). The incapability of pancreatic β-cells to produce adequate insulin or reduced insulin response in glucose deposit tissues leads to a chronic increase in blood glucose level followed by vascular damages which are the prime cause of death in diabetic patients (Bennett et al., 2018; Zheng et al., 2018). Previous studies in rodent models have shown that maternal nicotine treatment interrupts β-cell functions in offspring, and maternal SE induced intrauterine undernutrition is also associated with overconsumption in offspring, increasing the risk of insulin resistance and glucose intolerance (Holloway et al., 2005; Bruin et al., 2008, 2010; Sullivan et al., 2014). Therefore, to reduce the risk of metabolic disorder and diabetes, it is crucial to find effective preventive strategies to ameliorate the adverse impacts of maternal SE on offspring.

In the study of diabetes prevention, a high-protein diet has been proven to produce better glycemic control, fat loss, and preservation of muscle mass than a carbohydrate-rich diet with the same caloric intake (Skov et al., 1999; Parker et al., 2002; Layman et al., 2003). During this process, branch chain amino acids, especially L-leucine, have been suggested to play a critical role. Leucine is an essential amino acid and can only be obtained from the diet. In the brain, L-leucine activates the mammalian target of rapamycin in the hypothalamus (Cota et al., 2006), resulting in its beneficial effects of inducing weight loss, and improving glucose homeostasis in mouse models of obesity and diabetes (Arakawa et al., 2011; Chen et al., 2012; Westerterp-Plantenga et al., 2014). This evidence suggests that leucine supplement can be a potential strategy to prevent glucose disorders due to maternal SE.

The regulatory network for energy homeostasis has been widely studied in the hypothalamus where the neurons produce the appetite stimulator neuropeptide Y (NPY), and appetite suppressor α-melanocyte stimulating hormone cleaved from proopiomelanocortin (POMC) (Morton et al., 2006). Chemicals in cigarette smoke can inhibit hypothalamic NPY level and the same can happen in fetal brain and suckling pups when they continuously receive the chemicals from breastmilk. When the suckling pups are weaned, it can induce quitting smoking type of rebound response of NPY to cause overeating in childhood, similar to what happens to smokers when they quit smoking (Grove et al., 2001). In contrast, an intracerebroventricular administration of L-leucine, could reduce food intake and body weight in chow-fed animals, due to its suppression of hypothalamic NPY expression (Cota et al., 2006). Dietary leucine can pass the blood-brain barrier to access the brain (Chen et al., 2012); however, in the situation of high fat diet consumption, oral leucine supplement was not able to affect the adiposity but still can improve glycemic control (Chen et al., 2012).

While it is increasingly recognized the importance of the fetal environment on the susceptibility to future metabolic disorders, it is also important to look for effective early interventions to prevent adverse maternal impact in the offspring. Therefore, we hypothesized that in offspring from dams exposed to cigarette smoke during pregnancy, postnatal L-leucine supplement in drinking water will prevent excess fat accumulation and benefit glycemic control in adulthood. To address this hypothesis, we used our published mouse model of maternal SE (Huang et al., 2021) and supplied L-leucine in the drinking water to the female offspring from weaning for 10 weeks until they reached adulthood. As there is no sexual difference in metabolic disorders due to maternal SE, we only studied females. We aimed to investigate the effects of maternal SE and postnatal leucine supplement on body weight, adiposity, glucose tolerance, and the gene expression of metabolic markers in the hypothalamus and fat tissues.

## Materials and Methods

### Animal Experiments

According to the previous publication on the strain dependence of the response to cigarette smoke exposure (Vlahos et al., 2006), female Balb/C mice (6-week-age) were used. They were housed at 20 ± 2°C and maintained on a 12:12 h light/dark cycle (lights on 6:00 a.m.) with *ad libitum* access to standard rodent chow and water. All the animal experiments were approved by the Animal Ethics Committee of the San Yet-sun University (SYSU-IACUC-2020-81060) and followed the guidelines for animal care and use for scientific research by the National Institute of Health, USA.

After acclimatization, mice with similar body weight were randomly assigned to two groups, sham exposure (SHAM) and SE. The SE group was exposed to smoke produced by 2 cigarettes (Double Happiness; Tar: 8 mg; nicotine: 0.7 mg; CO: 12 mg) inside a perspex box (18 liters) in the fume hood, twice a day for 5 weeks before mating and during the gestation and lactation periods following a published protocol as we have previously published (Huang et al., 2021). The breeders in the SHAM group were treated identically except for the exposure to the air. After 5 weeks of preconditioning, females were placed with male mice to mate (ratio 3:1). All breeders continuously received the same treatments until pups were weaned. The pups and male breeders remained in their home cages when the females were treated and not subjected to any exposure.

### Post-weaning L-Leucine Supplement in Female Offspring

One day after birth, litter sizes were adjusted to 4–6 pups (sex ratio 1:1) to minimize the impact of milk competition. The pups were weaned at 21 days of age. Since there is no sexual difference in the metabolic disorders due to maternal SE, only female offspring were used in the present study (males were subjected to a different study). Within each litter, half of the pups were supplied with L-leucine (Sigma, St Louis, MO, USA) via drinking water (1.5% w/w) for 10 weeks. Normal drinking water was provided to the other half of the litter. This yielded four experimental groups, SHAM, SHAM-leucine, SE, and SE-leucine. Water intake was measured 1 week before the endpoint by recording the difference between the weight of the water bottle at a 24 h interval.

### Intraperitoneal Glucose Tolerance Test (IPGTT)

IPGTT was performed in the offspring at 12 weeks of age as previously described (Nguyen et al., 2015). In brief, after 5 h of fasting, the baseline of glucose level was measured in blood samples collected from the tail tip (T_0_) by Accu-Chek® glucose meter (Roche, Germany). After glucose injection (2 g/kg, ip), the same measurement was performed again at 15, 30, 60, and 90 min to calculate the area under the curve (AUC) for each mouse.

### Sample Collection

Female offspring were deeply anesthetized (ketamine/xylazine 180/32 mg/kg) at 13 weeks after overnight fasting. Blood glucose levels were immediately measured by Accu-Chek® glucose meter after blood collection. Plasma was stored at −20°C for the measurement of insulin levels using a commercially available ELISA kit following the manufacture's instruction (Abnova, Taiwan). The Homeostatic Model Assessment of Insulin Resistance (HOMA-IR) was calculated according to the formula: insulin (μU/mL) × glucose (mM)/22.5. Thereafter, mice were subsequently sacrificed by decapitation. The tissues including the hypothalamus, body fat (brown fat, epididymal, retroperitoneal, and visceral fat), organs (liver, kidney, and heart), as well as skeletal muscle (soleus, extensor digitorum longus (EDL), and quadratus lumborum) were dissected and weighed. Hypothalamus, retroperitoneal fat, and brown fat were stored at −80°C for subsequent measurement of mRNA expression of metabolic markers.

### Quantitative Real-Time PCR Assays

Total RNA was isolated from individual samples by TriZol Reagent following the manufacture's protocol [Invitrogen, United State of America (USA)]. The purified total RNA was used for First-strand cDNA generation with a synthesis kit (One-step gDNA Removal, TransGen Biotech, China). TaqMan probe/primers were pre-optimized and validated by the manufacturer (Thermo Fisher Scientific, USA) were used for quantitative real-time PCR (CFX96, Bio-Rad, USA). Target metabolic marker genes included neuropeptide Y (Npy: Mm00445771_m1), neuropeptide Y1 receptor (Npy1r: Mm00445771_m1), proopiomelanocortin (Pomc: Mm00435874_m1), melanocortin-4 receptor: (Mc4r: Mm00457483_s1), single minded gene 1 (Sim1: Mm00441390_m1), monocarboxylic acid transporters 2 (Mct2: Mm00441442_m1), monocarboxylic acid transporters 4 (Mct4: Mm01246824_g1), lactate dehydrogenase B (Ldhb: Mm05874166_g1) in hypothalamus and carnitine palmitoyl-transferase 1 alpha (Cpt1α:Mm00550438_m1), tumor necrosis factor alpha (Tnfα: Mm00443259_g1), adipose triglyceride lipase (Atgl: Mm01275939_g1), as well as uncoupling protein 1 (Ucp1: Mm00494069_m1) in BAT.

### Statistical Analysis

All the data are presented as mean ± SEM. Differences between the groups were analyzed using two-way ANOVA, except for glucose level during IPGTT which were analyzed by multi-factor ANOVA, followed by *post-hoc* LSD tests, if the data were normally distributed. If not, data were log-transformed to achieve normality of distribution before they were analyzed. *P* < 0.05 was considered statistically significant.

## Results

### Effects of Maternal SE and Postnatal L-Leucine Supplement on Anthropometry

At 13 weeks, mice in the SE groups showed smaller body weight than those in the SHAM group (*P* < 0.05, Table 1), consistent with the literature (Chan et al., 2016). Maternal SE also significantly reduced the weights of vital organs and skeletal muscle soleus (*P* < 0.05, SHAM vs. SE, Table 1). Although white fat masses also appeared smaller in the SE offspring, they did not reach statistical significance.

**Table 1:** Effects of maternal se and postnatal leucine supplement in female offspring.

**Offspring treatments**	**SHAM**	**SHAM-leucine**	**SE**	**SE-leucine**
	**(*n* = 18)**	**(*n* = 18)**	**(*n* = 16)**	**(*n* = 16)**
Body weight (g)	20.0 ± 0.3	19.3 ± 0.2^ψ^	18.6 ± 0.3^*^	18.4 ± 0.3^*^
Food intake (kJ/mouse/24 h)	34.9 ± 1.30	31.9 ± 1.76	36.7 ± 1.68	33.2 ± 1.93
HOMA	10.8 ± 1.21	9.07 ± 0.86	9.25 ± 0.93	10.5 ± 1.21
Naso-anal length (cm)	9.24 ± 0.08	9.17 ± 0.04	9.37 ± 0.37	8.96 ± 0.07
Liver (mg)	809 ± 14	712 ± 34^ψ^	745 ± 14	752 ± 21
Kidney (mg)	110 ± 2	107 ± 1	101 ± 3^*^	103 ± 2^*^
Heart (mg)	102 ± 3	99 ± 2	86 ± 3^*^	91 ± 2^*^
Brown fat (mg)	66.9 ± 2.5	62.7 ± 1.8	60.1 ± 3.0^*^	60.2 ± 1.8
Retroperitoneal fat (mg)	95.3 ± 7.6	76.3 ± 5.1^ψ^	80.9 ± 6.2	70.7 ± 5.6
Epididymal fat (mg)	521 ± 33	491 ± 22	507 ± 25	481 ± 36
Visceral fat (mg)	447 ± 16	428 ± 8	427 ± 14	403 ± 11
EDL (mg)	18.9 ± 0.6	18.7 ± 0.7	17.6 ± 0.4	16.8 ± 0.7^*^
Soleus (mg)	10.6 ±0.2	10.4 ± 0.2	9.8 ± 0.3^*^	9.9 ± 0.3
Quadra (mg)	253 ± 4	239 ± 7	242 ± 6	228 ± 8

*Results are expressed as mean ± SEM, and analyzed by two-way ANOVA, followed by post hoc LSD tests*.

* *P < 0.05, maternal SE effect*;

ψ *P < 0.05, leucine effect. EDL, extensor digitorum longus; Quadra, quadratus lumborum muscle; SE, smoke exposure*.

L-leucine supplement only significantly reduced body weight, liver weight, and retroperitoneal white fat mass in the SHAM offspring (P <0.05, SHAM-leucine vs. SHAM, Table 1). Such treatment did not significantly affect any of the anthropometric markers in the SE offspring, albeit smaller white fat mass (Table 1).

Neither maternal SE nor post-weaning L-leucine supplement affected food intake and somatic growth reflected by naso-anal length (Table 1). The water intake was similar between SHAM-leucine (3.62 ± 0.02 ml/mouse/day) and SE-leucine (3.17 ± 0.47 ml/mouse/day) groups in adulthood. This equivalent to a leucine intake of 54.3mg/mouse/day and 47.6mg/mouse/day at this time point.

### Leucine Supplement Normalized the Glucose Intolerance Caused by Maternal SE

**Figure 1:**
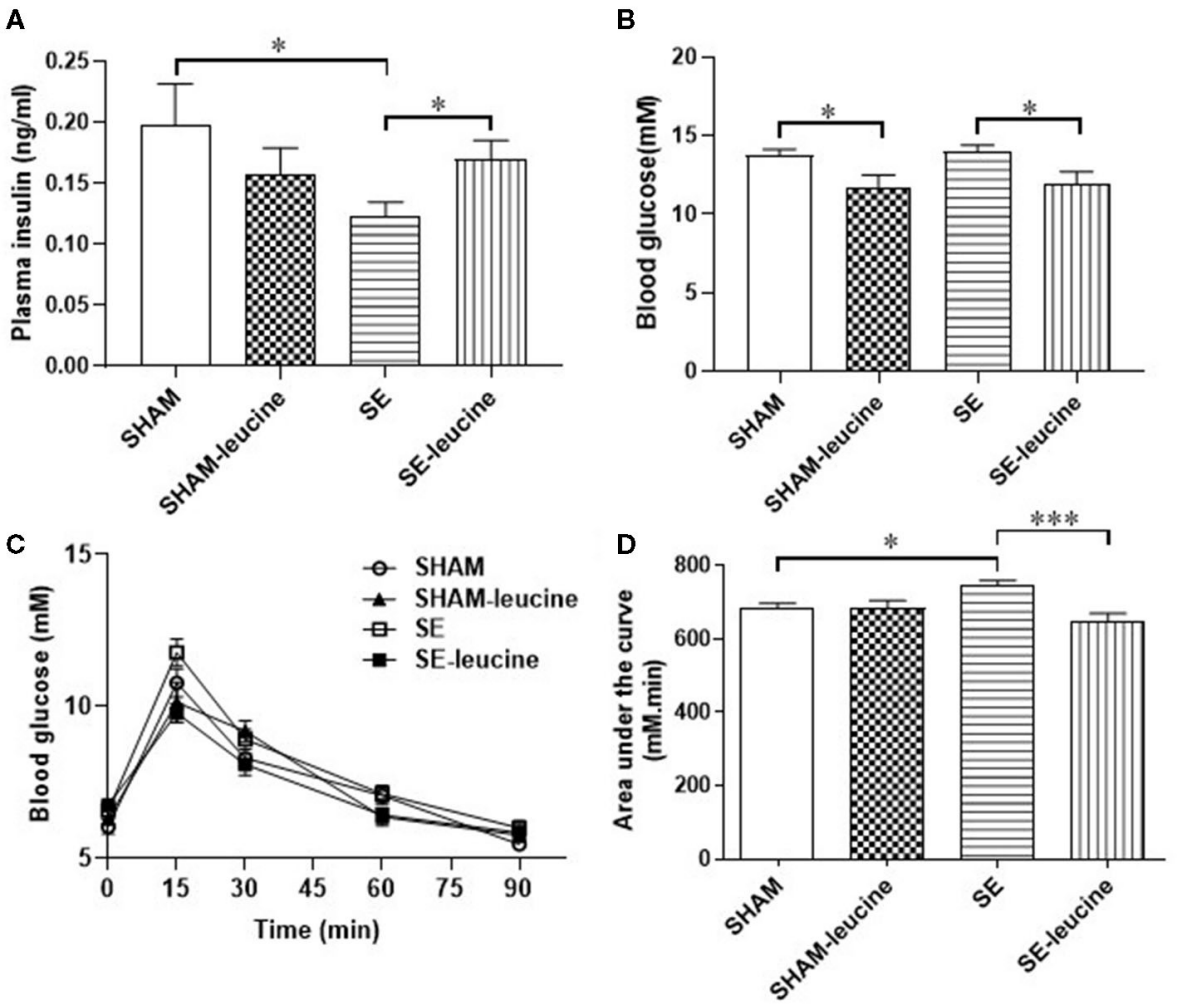
Blood glucose metabolic profile in female offspring. Plasma insulin **(A)** and blood glucose **(B)** level in female offspring at 13 weeks. Blood glucose levels during Intraperitoneal glucose tolerance test (IPGTT, **C**) and the area under the curve **(D)** at 12 weeks. Results are expressed as the mean ± SEM (*n* = 8), and analyzed by two-way ANOVA, followed by *post-hoc* LSD tests. *P < 0.05, ***P < 0.001.

At 13 weeks, plasma insulin level was significantly decreased in the SE group than that in the SHAM group (*P* < 0.05, SHAM vs. SE, Figure 1A), although fasting blood glucose levels were similar between the groups (Figure 1B). In addition, adult SE offspring exhibited impaired ability to clear blood glucose during IPGTT compared with the SHAM offspring, reflected by the higher AUC values (*P* < 0.05, SHAM vs. SE, Figures 1C,D).

The supplement of leucine normalized plasma insulin level in the SE offspring (*P* < 0.05, SE-leucine vs), reduced fasting blood glucose in both SHAM and SE offspring (*P* < 0.05 vs. untreated littermates, Figure 1B). Moreover, leucine supplement effectively normalized glucose tolerance in the SE offspring (*P* < 0.001, SE vs. SE-leucine, Figure 1D). However, systematic glucose metabolism seems not to be related to insulin resistance, as HOMA index was not significantly changed by maternal SE or leucine supplement (Table 1).

### L-Leucine Supplement Restored *Pomc* Expression in the Hypothalamus of SE Offspring

**Figure 2:**
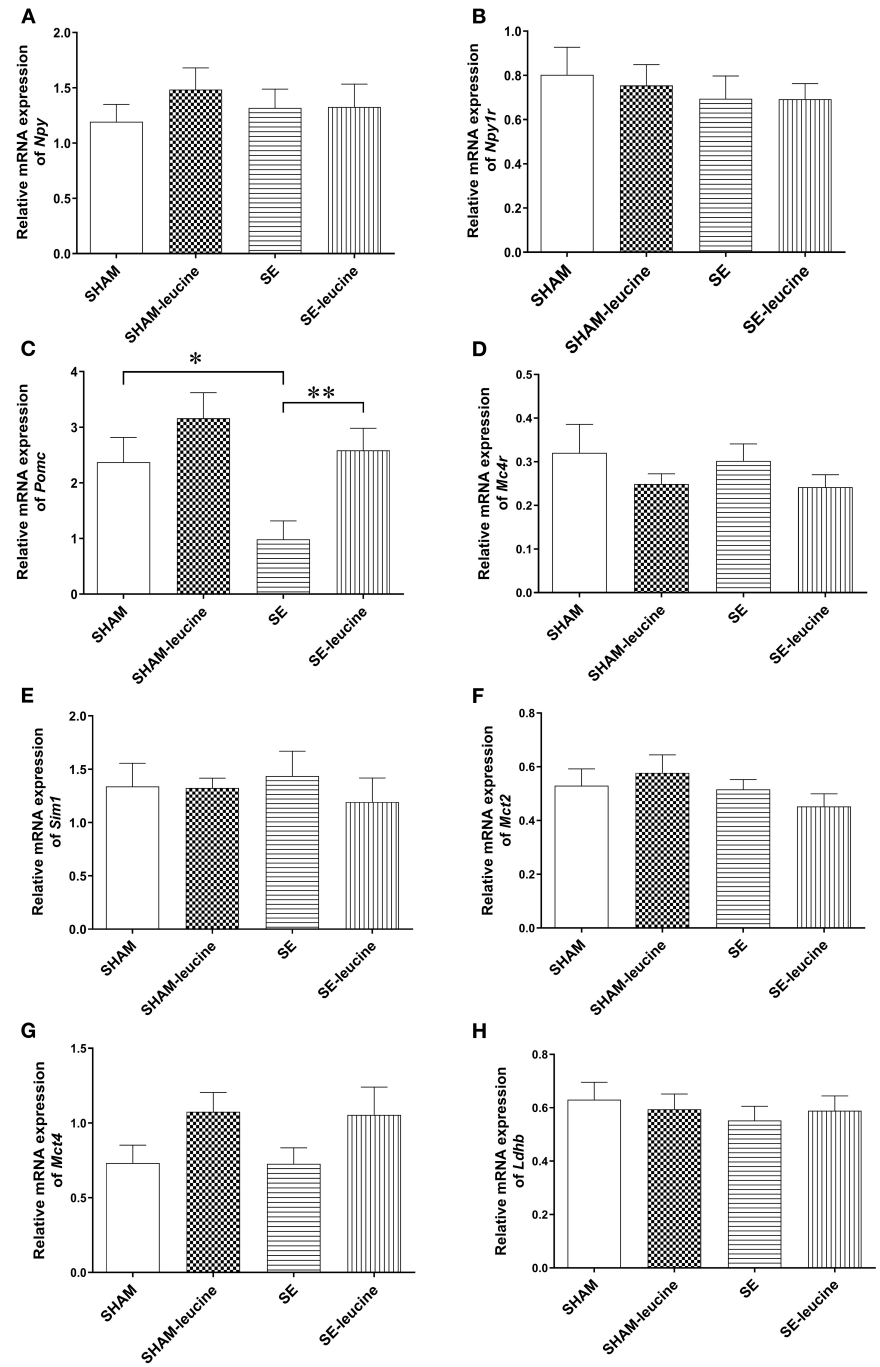
Expression of metabolic regulators in the hypothalamus. The mRNA expression of *Npy* **(A)**, *Npy1r* **(B)**, *Pomc* **(C)**, *Mc4R* **(D)**, *Sim1* **(E)**, *Mct2* **(F)**, *Mct4* **(G)**, and *Ldhb* **(H)** in the hypothalamus of female offspring at 13 weeks (*n* = 8). Results are expressed as mean ± SEM and analyzed by two-way ANOVA, followed by *post hoc* LSD tests. *P < 0.05, **P < 0.01.

To evaluate the effects of the maternal SE and post-weaning leucine supplement on the feeding regulators, we measured food intake and analyzed the mRNA expression of classical genes involved in appetite regulation in the hypothalamus. There was no difference in food intake among the groups (Table 1). In the SE offspring, the levels of hypothalamic *Npy* and *Npy1r* mRNA expression were similar to those in the SHAM offspring (Figures 2A,B), whereas the *Pomc* expression was markedly downregulated (*P* < 0.05, SHAM vs. SE, Figure 2C). However, the expression of *Mc4r*, which is the dominant anorexigenic receptor for Pomc derived α-melanocyte-stimulating hormone, its downstream signaling *Sim1*, as well as lactate transports *Mct2* and *Mc4* and *Ldhd* (involved in lipid metabolism) was not affected by maternal SE (Figures 2D–H), in line with unchanged food intake.

Postweaning supplement of L-leucine only normalized *Pomc* expression in SE-leucine group (*p* < 0.01, SE vs. SE-leucine, Figure 2C). It did not significantly change any of the other metabolic regulators in the hypothalamus measured in this study, although there is a trend in increased *Mct4* in both SHAM and SE offspring (~50% increase compared with their untreated littermates, Figure 2G). However, food intake was not significantly changed by leucine supplement (Table 1).

### Effects on the Substrate Metabolic in the Fat in Female Offspring

**Figure 3:**
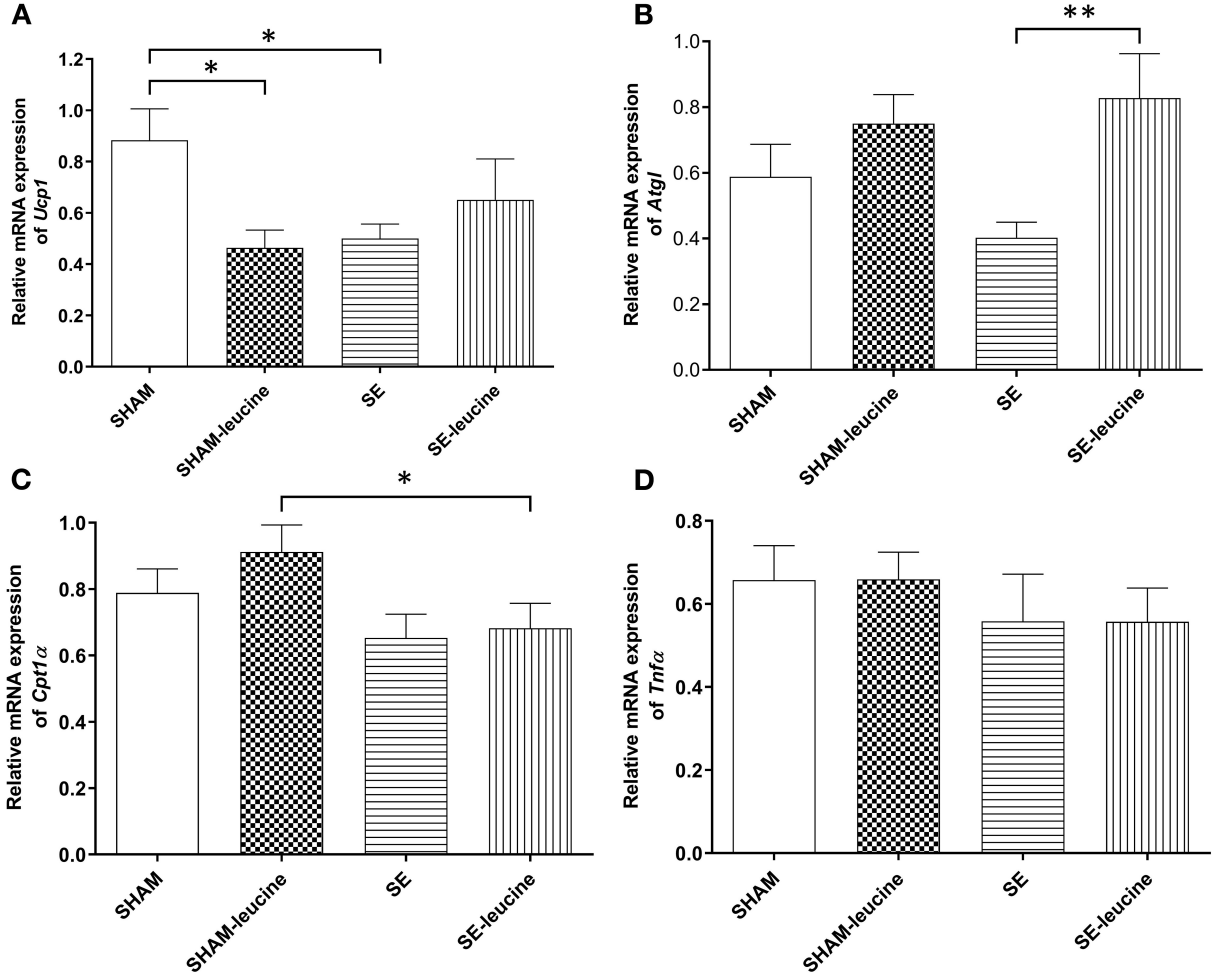
Expression of lipid metabolic markers in the retroperitoneal white fat tissue. mRNA expression of uncoupling protein (Ucp)1 in the brown fat **(A)**, *Atgl* **(B)**, *Cpt1*α **(C)**, and *Tnf* α **(D)** in the retroperitoneal fat, in female offspring at 13 weeks (*n* = 8). Results are expressed as mean ± SEM. Data were analyzed by two-way ANOVA, followed by *post-hoc* LSD tests. *P < 0.05, **P < 0.01.

In the SE group, the expression of the thermogenesis markers *Ucp1* was downregulated by maternal SE (*P* < 0.05, Figure 3A). There was a trend decrease in the expression of *Atgl* in retroperitoneal fat by maternal, but the difference was not statistically significant (Figure 3B). The expression of *Cpt1*α, a rate-limiting regulator for fatty acid β-oxidation in mitochondria, and inflammatory marker *Tnf*α was not affected by maternal SE (Figures 3C,D).

L-leucine supplement reduced *Ucp1* expression only in the SHAM offspring (*P* < 0.05, SHAM-leucine vs. SHAM), and increased *Atgl* expression only in the SE offspring (*P* < 0.01, Figure 3B). Interestingly, although leucine showed no impact on *Cpt1*α expression in either SHAM or SE offspring, its level was significantly lower in SE-leucine mice compared to SHAM-leucine group (*P* < 0.05, SHAM-leucine vs. SE-leucine, Figure 3A). The expression of *Tnf*α was not affected by L-leucine supplement (Figure 3D).

## Discussion

The smoking cessation rate among pregnant women is quite low (Coleman et al., 2015). This possesses a significant health risk in their offspring later in life. Finding a non-invasive and effective prophylactic strategy to protect such offspring can significantly improve their quality of life in the long term. The novel approach of this study is the introduction of post-weaning leucine supplement in the offspring to ameliorate the adverse impact of maternal SE. We found that leucine supplement normalized glucose metabolic disorders and insulin insufficiency caused by maternal SE in adult offspring. Even in the SHAM offspring, leucine supplement reduced their fat mass and lowered fasting blood glucose without affecting somatic growth, suggesting its benefit to metabolic profile in general.

In this study, we demonstrated the detrimental effect of maternal SE during pregnancy, which led to smaller body weight, glucose intolerance, and lower plasma insulin level in adult offspring. This is consistent with the literature (Saad et al., 2018; Li et al., 2019). Our study also showed long-lasting effects of maternal SE during pregnancy on reduced lean body mass, including the vital organs (e.g., liver) and skeletal muscle soleus. Tobacco smoke contains over 5000 harmful chemicals (Talhout et al., 2011). The nicotine in cigarette smoke was found to reduce the uteroplacental blood flow leading to inefficient nutrition supplement for the growing fetus. Nicotine can also cross the blood-placental barrier to enter the fetal circulation to concentrate higher nicotine level than that in smoking mother, which further disrupted normal development of the endocrine, neurologic, respiratory, and cardiovascular systems (Lisboa et al., 2012; Holbrook, 2016; Jamshed et al., 2020). In addition, CO inhibits oxygen delivery to the cells and cause *in utero* hypoxia due to a great affinity to hemoglobin and increases carboxyhemoglobin levels in umbilical arteries (Ko et al., 2014). In the present study, fetal underdevelopment was not corrected by so called “catch-up growth” after birth in humans (Al Mamun et al., 2006; Darendeliler, 2019). This may be due to the diet in the animal study, which is standard rodent chow with balanced nutrition; whereas in humans, smoking mothers are more likely to consume foods high in sugar and saturated fat which they also feed to their children (Al Mamun et al., 2006). This dietary choice would encourage the development of obesity in such offspring. Future studies can introduce the second insult of high fat diet consumption in the offspring to further study the mechanism underlying metabolic disorders in this population.

Furthermore, we also observed that maternal SE would induce glucose intolerance together with reduced plasma insulin level, which is consistent with the previous study in both human and mouse models (Henkin et al., 1999; Chen et al., 2011). In rodent models of nicotine exposure during lactation, pancreatic β-cells depletion with subsequent impaired glucose homeostasis was observed, suggesting the toxic effect of nicotine on β-cell function (Bruin et al., 2010; Primo et al., 2013). In addition, in the major glucose deposition organ fat tissue, the pro-inflammatory cytokine TNFα can directly block the insulin receptor signaling pathway to cause insulin resistance and glucose intolerance (Montgomery and Ekbom, 2002; Vuguin et al., 2004). However, in this study, the expression of fat *Tnf*α was not changed by maternal SE, in line with HOMA index ruling out the involvement of insulin resistance, suggesting insufficient insulin production may be the major driver of glucose metabolism disorder in the offspring with *in-utero* SE exposure. In humans, maternal smoking is linked to an increased risk of type 2 diabetes independent of diet choices (Jaddoe et al., 2014).

In the SE offspring, the fat mass of all pads collected in this study was reduced although without statistical significance. This may lead to the adaptive reduction in *Ucp1* expression. UCP1 mediate non-shivering thermogenesis and basal metabolic rate in brown fat (Cannon and Nedergaard, 2004). Fasting or chronic food restriction can downregulate *Ucp1* expression (Cannon and Nedergaard, 2004; Oelkrug et al., 2015; Peixoto et al., 2020). It is unclear the contribution of downregulated hypothalamic *Pomc* expression as none of its downstream signaling elements in the regulation of metabolic homeostasis was changed accordingly, which requires investigations into other functions of *Pomc*. It may also be an adaptive response to reduced fat mass.

L-leucine supplements have been shown to improve glucose metabolism and insulin sensitivity even in obese rats (Bassil et al., 2007; Berry-Kravis et al., 2007; Chen et al., 2012). Indeed, the most prominent benefit of leucine supplement is perhaps the normalized glucose metabolism in the SE offspring. Since HOMA index was not significantly affected, the mechanism of improved glucose metabolism may not be related to insulin sensitivity, but driven by significantly increased blood insulin levels. This observation is supported by previous studies showing the beneficial effect of leucine on β-cell function and metabolism (Yang et al., 2010). L-leucine is an essential amino acid that is normally obtained via diet. Considering the independent risk of type 2 diabetes by maternal smoking, human trials can be considered to implement such prophylactic treatment.

Furthermore, in the SHAM offspring, L-leucine administration reduced their body weight and fat mass. As a result, *Ucp1* is downregulated to prevent excessive energy expenditure. Previous studies in mice demonstrated that leucine promotes fat loss by increasing fatty acid utilization in adipocytes (Gual et al., 2005; Sun and Zemel, 2007; Zhang et al., 2007). Atgl is an essential enzyme to break down triglycerides into free fatty acids and glycerol and CPT1α is the rate limiting step for β-oxidation of fatty acids into ATP in the mitochondria. With L-leucine supplement, both markers were non-significantly increased in the SHAM offspring. It raised the question of whether a statistical significance is required to exert physiological function. Nevertheless, the administration of leucine upregulated *Atgl* expression, in the face of some reduction in fat mass. In the SE offspring, fat loss was not as prominent as the SHAM offspring. This may be due to the need for a minimum amount of fat tissue to maintain normal metabolic function. Leucine did not show a measurable impact on the key metabolic regulators in the hypothalamus, which is somewhat consistent with our previous study using long-term leucine supplement (Chen et al., 2012). This suggests that peripheral tissues may be the primary targets in such setting. Future studies can also investigate whether this alteration can protect the SE offspring from high fat diet consumption induced obesity.

We need to acknowledge the limitations of this study. We only trialed one dose of L-leucine according to our previous study (Chen et al., 2012) in the female offspring to prove the concept. Previously, leucine supplement was shown to improve glucose metabolic disorders due to maternal obesity in male offspring (Chen et al., 2012; Saad et al., 2018). As this study was only performed on female mice, the positive outcome on glucose metabolism may not happen in the male offspring, who also showed similar metabolic disorders in our previous studies on the same model (Nguyen et al., 2015; Saad et al., 2018; Li et al., 2019; Huang et al., 2021). Future studies can investigate a dose-response curve of such treatment in both male and female offspring. In this study, we only measured the classical metabolic regulators, which were not affected by a combination of a nutritional balanced diet and leucine. This may be due to glucose intolerance as the only measurable abnormality in this model. Future studies can induce more severe metabolic disorders by introducing additional risk factors.

## Conclusion

L-leucine supplement can reduce fat mass potentially by increasing lipid breakdown and/or metabolism in the fat tissue in both SHAM and SE offspring. Importantly, our study also suggests the benefits of postnatal L-leucine supplement to improve glucose tolerance in SE offspring by improving their blood insulin levels, which may potentially reduce the risk of type 2 diabetes. Future studies can employ additional high fat diet feeding in the SE offspring to confirm the prophylactic effects of L-leucine supplement on the development of obesity and type 2 diabetes.

## Data Availability Statement

The raw data supporting the conclusions of this article will be made available by the authors, with valid justification.

## Ethics Statement

The animal study was reviewed and approved by Animal Experimentation Ethics Committee of the San Yet-sun University (number: SYSU-IACUC-2020-81060).

## Author Contributions

YZ: methodology, investigation, and writing—original draft. TH: investigation and editing. NW, YX, CS, and MH: investigation. BO: conceptualization, supervision, and review. CY: conceptualization, supervision, writing, and editing. HC: conceptualization, supervision, writing, and review. All authors contributed to the article and approved the submitted version.

## Conflict of Interest

The authors declare that the research was conducted in the absence of any commercial or financial relationships that could be construed as a potential conflict of interest.
